# MicroRNA expression profiles in human cancer cells after ionizing radiation

**DOI:** 10.1186/1748-717X-6-29

**Published:** 2011-03-31

**Authors:** Olivier M Niemoeller, Maximilian Niyazi, Stefanie Corradini, Franz Zehentmayr, Minglun Li, Kirsten Lauber, Claus Belka

**Affiliations:** 1 Department of Radiation Oncology, Ludwig-Maximilians University of Munich, Germany

## Abstract

**Introduction:**

MicroRNAs are regulators of central cellular processes and are implicated in the pathogenesis and prognosis of human cancers. MicroRNAs also modulate responses to anti-cancer therapy. In the context of radiation oncology microRNAs were found to modulate cell death and proliferation after irradiation. However, changes in microRNA expression profiles in response to irradiation have not been comprehensively analyzed so far. The present study's intend is to present a broad screen of changes in microRNA expression following irradiation of different malignant cell lines.

**Materials and methods:**

1100 microRNAs (Sanger miRBase release version 14.0) were analyzed in six malignant cell lines following irradiation with clinically relevant doses of 2.0 Gy. MicroRNA levels 6 hours after irradiation were compared to microRNA levels in non-irradiated cells using the "Geniom Biochip MPEA homo sapiens".

**Results:**

Hierarchical clustering analysis revealed a pattern, which significantly (p = 0.014) discerned irradiated from non-irradiated cells. The expression levels of a number of microRNAs known to be involved in the regulation of cellular processes like apoptosis, proliferation, invasion, local immune response and radioresistance (e. g. miR-1285, miR-24-1, miR-151-5p, let-7i) displayed 2 - 3-fold changes after irradiation. Moreover, several microRNAs previously not known to be radiation-responsive were discovered.

**Conclusion:**

Ionizing radiation induced significant changes in microRNA expression profiles in 3 glioma and 3 squamous cell carcinoma cell lines. The functional relevance of these changes is not addressed but should by analyzed by future work especially focusing on clinically relevant endpoints like radiation induced cell death, proliferation, migration and metastasis.

## Introduction

MicroRNAs are small non-coding RNAs of typically 20 - 22 base pairs length. They are involved in gene regulation at the post-transcriptional level by silencing mRNA translation. To date more than 1000 microRNAs have been discovered. MicroRNAs are involved in the regulation of diverse cellular processes, including programmed cell death, proliferation, differentiation, metabolism, migration and stress responses (for review see [1]). Notably, a single microRNA potentially regulates a wide range of target genes resulting in a global impact on gene expression [2].

As central regulators of gene expression, microRNAs have been implicated in the pathogenesis of human cancers, acting either as tumor suppressors [3,4] or as oncogenes [5]. In fact, certain microRNA profiles of human cancers have been found to correlate with the malignant phenotype of cancer cells when compared to normal cells (for review see [6]). Clinically important, the expression of distinct microRNAs seems to be associated with the prognosis [7] and may also predict the efficacy of therapeutic interventions, including radiotherapy [8,9]. In fact, microRNAs have been shown to modulate the radiosensitivity of lung cancer cells *in vitro *[10] and breast cancer cells *in vivo *[11]. Moreover, normal cells show altered levels of microRNAs in response to ionizing radiation [12,13].

The response of cancer cells to ionizing radiation has been extensively studied resulting in the discovery of central regulators of radiosensitivity. However, irradiation-induced alterations in microRNA profiles have hitherto not been analyzed. This stimulated us to perform the present study: a microarray based analysis of irradiation-induced changes in all microRNAs published in the Sanger miRBase release version 14.0 (see http://microrna.sanger.ac.uk/sequences/index.shtml). We describe alterations in the abundance of 1100 microRNAs in six malignant cell lines following irradiation. To our knowledge this represents the broadest analysis of irradiation induced changes in microRNA patterns to date.

## Materials and methods

### Cell culture

For the analysis of characteristic microRNA patterns, six different cell lines were used: Squamous cell carcinoma of the head and neck (SCC-4, SCC-25, CAL-27) and cell lines from brain tumors (LN229, T98G, U-87 MG). SCC-4, SCC-25 and CAL-27 were purchased from the "Deutsche Sammlung von Mikroorganismen und Zellkulturen" (DSMZ, Braunschweig Germany), T98G and U-87 MG were purchased from the European Collection of Cell Cultures (ECACC, UK). LN229 was a gift from the Department of Neurosurgery, University of Munich. SCC-4 and SCC-25 were grown in Dulbecco's MEM/Ham's F-12 medium supplemented with 20% fetal bovine serum (FBS) and Hydrocortisone (40 ng/ml) and Sodium Pyruvate (1 mM), respectively. CAL-27 were grown in Dulbecco's MEM, supplemented with 10% FBS. LN229, T98G, U-87 MG were grown in Earles buffered Salt Solution (EBSS), supplemented with 10% FBS, Sodium Glutamine (2 mM), Non Essential Amino Acids (1x) and Sodium Pyruvate (1 mM). All media and supplements were purchased from Biochrom, Germany.

### Irradiation

Cells were seeded in culture flasks and grown for 5 - 10 passages. For the experiments, cells were grown to a confluency of ~70%. Cells were then irradiated with 6 MeV Photons at a dose rate of 3 Gy per minute using a linear accelerator (Siemens Mevatron) to a total dose of 2 Gy. After irradiation cells were incubated at 37°C for 6 hours before extraction of the total RNA. Non-irradiated cells were used as controls.

### Isolation of total RNA

Total RNA was extracted using the *mir*Vana™ microRNA Isolation Kit (Ambion) according to the manufacturer's instructions. Quantity and quality of the extracted RNA was analyzed with the Agilent 2100 Bioanalyzer (Agilent Technologies), using the company's RNA 6000 Nano Kit according to the manufacturer's instructions. RNA extracts were stored at - 20°C.

### Analysis of the microRNAs

The RNAs patterns were analyzed by Febit (Heidelberg) using the company's "Geniom Biochip MPEA homo sapiens", generating five data points for each microRNA measured. To adjust for a systematic spatial variability on each microarray, the intensities of black probes were used for background correction. To test the hybridization process as well as positioning features additional hybridization controls were added to the array template (data not shown).

### Statistical analysis

Hierarchical cluster analysis (bottom-up complete linkage clustering using Euclidean distance as a measure) was performed using the normalized data of the 65 most deregulated microRNAs to identify differentially expressed microRNAs following irradiation. The correlation between two dichotomous variables was assessed using the chi-square test. A two-tailed p-value < 0.05 was considered significant. MicroRNA changes in single cell lines were not analyzed because the statistical power was not sufficient.

## Results

**Figure 1:**
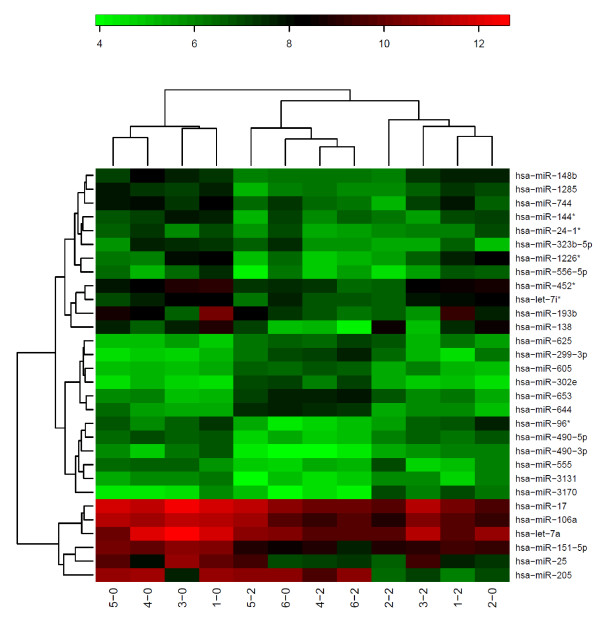
**Heatmap and clustering of samples and probes for non-irradiated versus irradiated cells**. The first number indicates the cell line (1 = T98G, 2 = U-87 MG, 3 = LN229, 4 = SCC-25, 5 = SCC-4, 6 = CAL-27) the second number indicates irradiation of the cell line (0 = non-irradiated; 2 = irradiated). On the left side, most of the non-irradiated cell lines cluster together while on the right side most of the irradiated cell lines form a cluster.

Hierarchical cluster analysis revealed two clusters of microRNAs which significantly (p = 0,014) differentiated between irradiated and non-irradiated cells. Figure 1 shows the heatmap comparing irradiated and non-irradiated cells.

Although statistical interpretation of the data is difficult since commonly used p-values of 0.05 might lead to false positive results when analyzing more than 1000 microRNAs, the microRNAs displaying the most striking up- or downregulation after irradiation are presented in Table 1.

**Table 1:** Most deregulated microRNAs in six malignant cell lines following irradiation

microRNA	Fold change	Log2-value	p-value (unadjusted)
miR-24-1*	3.07	1.12	0.01

miR-144*	2.93	1.07	0.01

miR-1285	3.04	1.11	0.04

miR-490-5p	2.98	1.09	0.04

miR-151-5p	3.33	1.20	0.04

let-7i*	2.97	1.09	0.04

miR-3131	3.11	1.14	0.06

miR-323b-5p	4.75	1.56	0.06

miR-625	0.34	-1.08	0.06

miR-744	3.10	1.13	0.07

miR-605	0.31	-1.17	0.07

miR-106a	4.22	1.44	0.08

miR-148b	3.23	1.17	0.09

miR-1226*	5.81	1.76	0.10

miR-452*	2.89	1.06	0.10

let-7a	4.60	1.53	0.10

miR-556-5p	3.08	1.12	0.11

miR-17	4.38	1.48	0.11

miR-96*	3.24	1.18	0.13

miR-653	0.28	-1.28	0.16

miR-490-3p	2.88	1.06	0.20

miR-299-3p	0.27	-1.31	0.21

miR-25	3.67	1.30	0.23

miR-3170	0.32	-1.14	0.23

miR-205	9.66	2.27	0.26

miR-555	3.13	1.14	0.28

miR-138	4.15	1.42	0.29

miR-193b	3.71	1.31	0.36

miR-302e	0.34	-1.08	0.44

Unadjusted p-values are presented.

When comparing irradiated cells with their non-irradiated counterparts, the levels of several microRNAs that target central regulators of cancer cells were found to be susceptible to ionizing radiation. Of note, miR-1285, which negatively regulates the expression of the crucial tumor suppressor p53 [14], was upregulated approximately 3-fold (p = 0.02, unadjusted). MiR-151-5p known to enhance migration and metastasis in human hepatocellular carcinoma [15] was another microRNA, whose level was found to be upregulated approximately 3-fold, thus potentially hampering the therapeutic effort.

On the contrary, miR-24-1, a member of the miR23b cluster [16] that interferes with Transforming Growth Factor β (TGFβ) expression, was also up-regulated approximately 3-fold (p = 0.0048, unadjusted). Elevated TGFβ expression in human malignancies has been reported to be associated with enhanced angiogenesis, local immune suppression and increased invasiveness (for review see [17]). Let-7i, a member of the let7-family negatively modulating tumor growth in human cancers [18] was also upregulated approximately 3-fold (p = 0.03, unadjusted). Notably, let-7i and its family member let-7a were the only microRNAs found in our screen that had already been described to be up-regulated following irradiation.

Moreover, our screen identified different other microRNAs with so far unknown function to be up- or downregulated in response to irradiation. For many of them this is first time that they are reported to be susceptible to irradiation, like for instance, miR-144*, which was upregulated approximately 3-fold (p = 0.0026, unadjusted). A detailed comparison of the levels of all 1100 microRNAs before and after irradiation can be found in the additional file 1.

## Discussion

The data presented here show that ionizing radiation with clinically relevant doses of 2 Gy stimulate significant changes in microRNA expression patterns in different cancer cell lines. These changes might well influence the clinical outcome, since the expression of microRNAs, which are known to regulate apoptosis, migration and proliferation was altered in response to irradiation.

Although the functional role of single microRNAs can not be established from the data presented here, several hypotheses can by generated which should be addressed by future experiments.

In this context, the up-regulation of the Epidermal Growth Factor Receptor (EGF-R) following ionizing radiation might serve as a paradigm. Irradiation-induced EGF-R expression leads to increased radioresistance of malignant cells in subsequent treatment sessions during the course of fractionated radiotherapy [19]. Similarly, an increased expression of mirR-1285, a negative regulator of the cardinal tumor suppressor p53, as it was observed in the present study might possibly lead to increased radioresistance in subsequent radiotherapy sessions. Furthermore, irradiation-induced changes in microRNA expression levels might also affect migration and metastasis of surviving cells. In this context, ionizing radiation-induced over-expression of miR-151-5p as described here might enhance dissemination and migration of malignant cells during a course of radiation therapy, since miR151-5p was found to increase migration and intra-hepatic metastasis in hepatocellular carcinoma [15]. Indeed, enhanced migration of malignant glioma cells was observed in response to radiotherapy [20,21].

Another candidate for regulating responsiveness to anticancer therapy is the let-7 family, although certain members of the let-7 family had different effects on radiation sensitivity in A549 lung cancer cells [10]. Let-7 family members are down-regulated in lung cancer cells [9] which possibly increases cell growth by increasing KRAS levels [22]. Over-expression of let-7a, which in the present study was up-regulated 4,6-fold (n. s.), was shown to increase radiation sensitivity in lung cancer cells [23]. Moreover, low levels of let-7a correlated with poor survival in patients with lung cancer [8,9], while over-expression of let-7a inhibited growth of lung cancer cells in vitro [9]. On the other hand, let-7i, which in the present study was observed to be up-regulated following irradiation, might increase growth, since it was shown that decreased levels of let-7i decreased growth of malignant cells and increased drug potency [18].

Another mechanism, through which ionizing radiation exerts its effects, involves changes in the tumor microenvironment. Interestingly, miR-24-1 levels were increased following irradiation and miR-24-1 might influence angiogenesis, invasion and local immune response through down-regulation of TGFβ [16].

In summary, the present study revealed altered expression levels of microRNAs known to influence apoptosis, migration and proliferation, angiogenesis and local immune response in response to irradiation. Moreover, a number of microRNAs with unknown functions were found to be radiation-responsive.

The power of the present study is based upon the huge number of investigated microRNAs (>1000) and the combined analysis of different malignant cells lines. Although adjusted p-values of changes in the expression levels of single microRNAs were not significant because of the huge number of microRNAs analyzed, the changes observed allow the generation of hypotheses and the design of further experiments validating the initial findings presented here and investigating the functional relevance of microRNA level alterations in the context of radiation oncology.

## Competing interests

The authors declare that they have no competing interests.

## Authors' contributions

OMN: RNA-Isolation and Purification, writing Manuscript, MN: Statistics and critical revision of the manuscript, SC: Cell culture and critical revision of the manuscript, FZ: Organization and Negotiation with Febit and critical revision of the manuscript, ML: Irradiation and critical revision of the manuscript, KL: Support concerning all technical questions, planning of experiments and critical revision of the manuscript.

CB: Development of the concept and critical revision of the manuscript.

All authors read and approved the final manuscript.

## Supplementary Material

Additional file 1 **Changes in 1100 microRNAs of six malignant cell lines following irradiation**. Raw data of the levels of all 1100 microRNAs before and after irradiation. Click here for file
